# Supplementary material for: High-throughput Discovery of Topologically Non-trivial Materials using Spin-orbit Spillage
Source: Sci Rep. 2019 Jun 12;9:8534. doi: 10.1038/s41598-019-45028-y (PMC6561936; doi:10.1038/s41598-019-45028-y)
Supplement: Supplementary file 1 — Supplementary information: High-throughput Discovery of Topologically Non-trivial Materials using Spin-orbit Spillage [file 41598_2019_45028_MOESM1_ESM.docx]

**Supplementary information: High-throughput Discovery of Topologically Non-trivial Materials using Spin-orbit Spillage**

Kamal Choudhary, Kevin F. Garrity, Francesca Tavazza

1 Materials Science and Engineering Division, National Institute of Standards and Technology,

Gaithersburg, Maryland 20899, USA


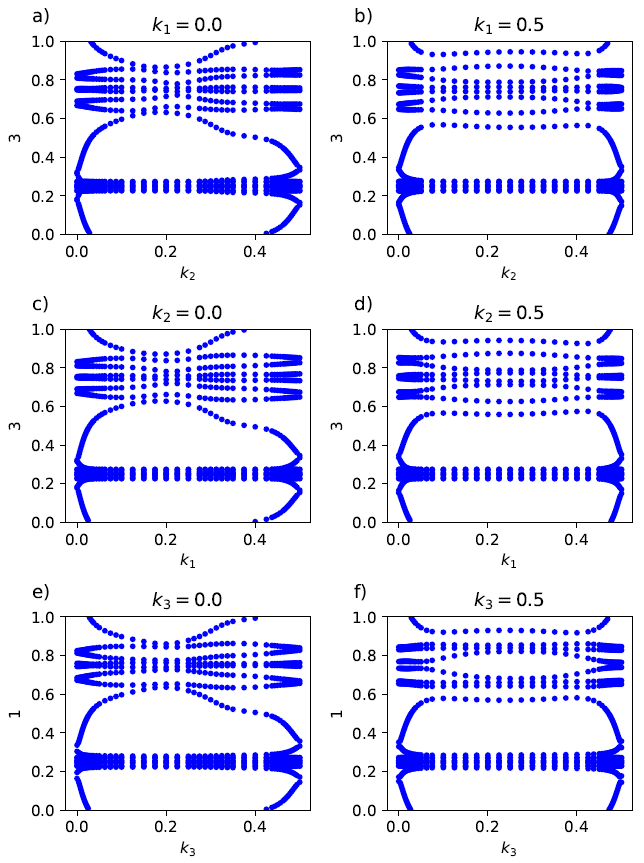


*Fig. S1 Wannier-charge center plot for Ba_2_HgPb along different surfaces.*


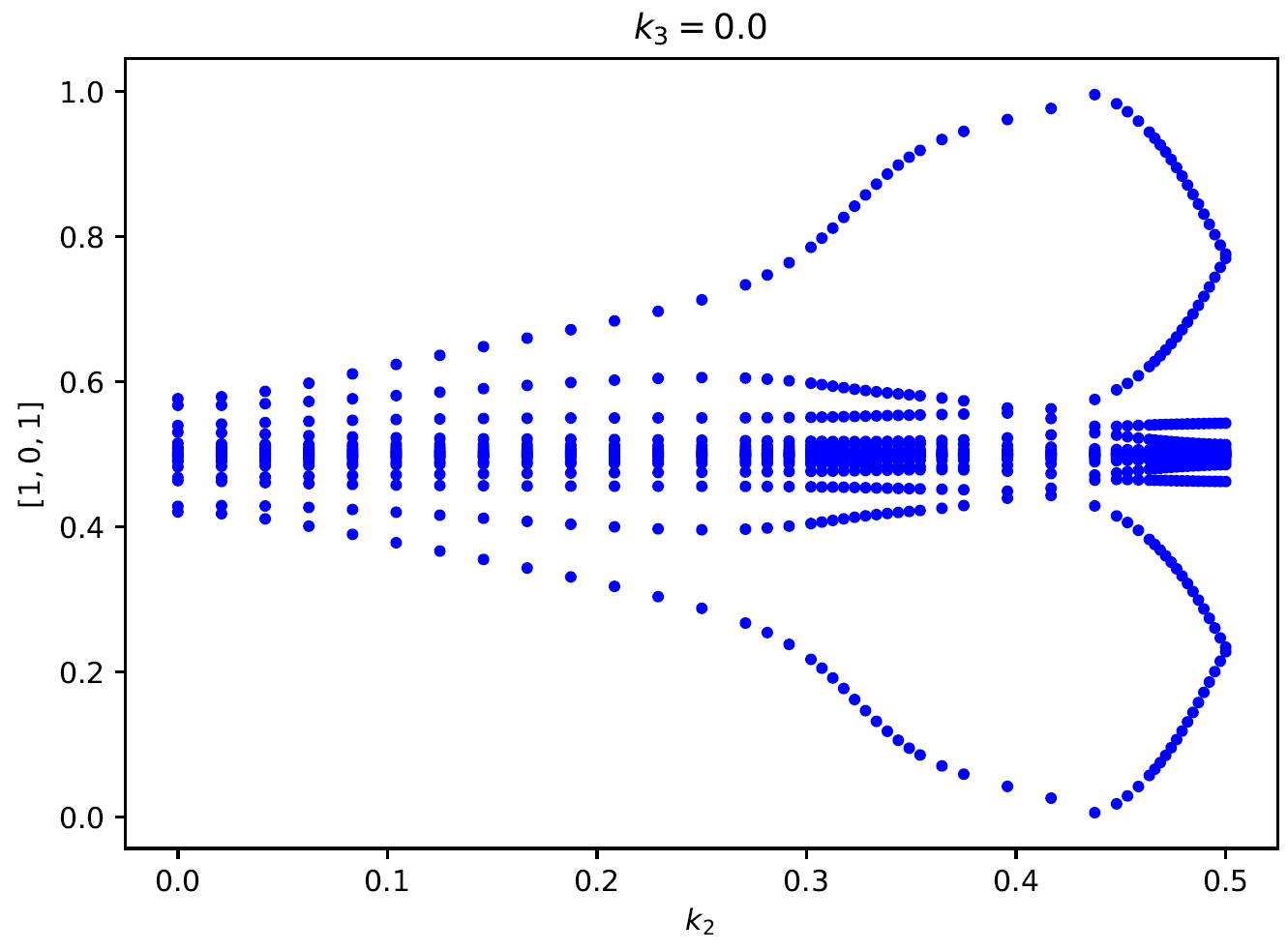


*Fig. S2 Wannier-charge center plot for Ba_2_HgPb along [1,0,1]. The plot shows gapless charge centers which is an indication of non-trivial behavior of the material.*


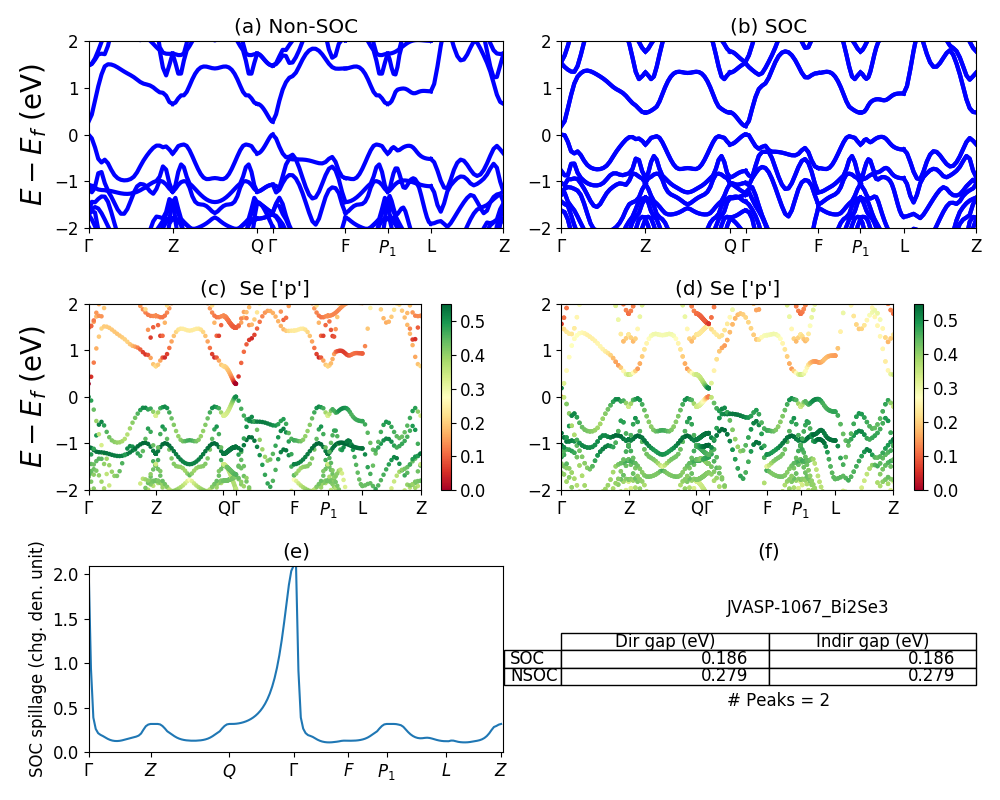


*Fig. S3 a) Non-SOC and b) SOC band structures of Bi_2_Se_3_ (JVASP-1067,* *https://www.ctcms.nist.gov/~knc6/jsmol/JVASP-1067). c) non-SOC and d) SOC projected band structures, projecting onto Se-p orbitals. e) Spillage, as a function of k. f) Table of bandgaps and spillage information. Bi_2_Se_3_ is an example topological insulator.*


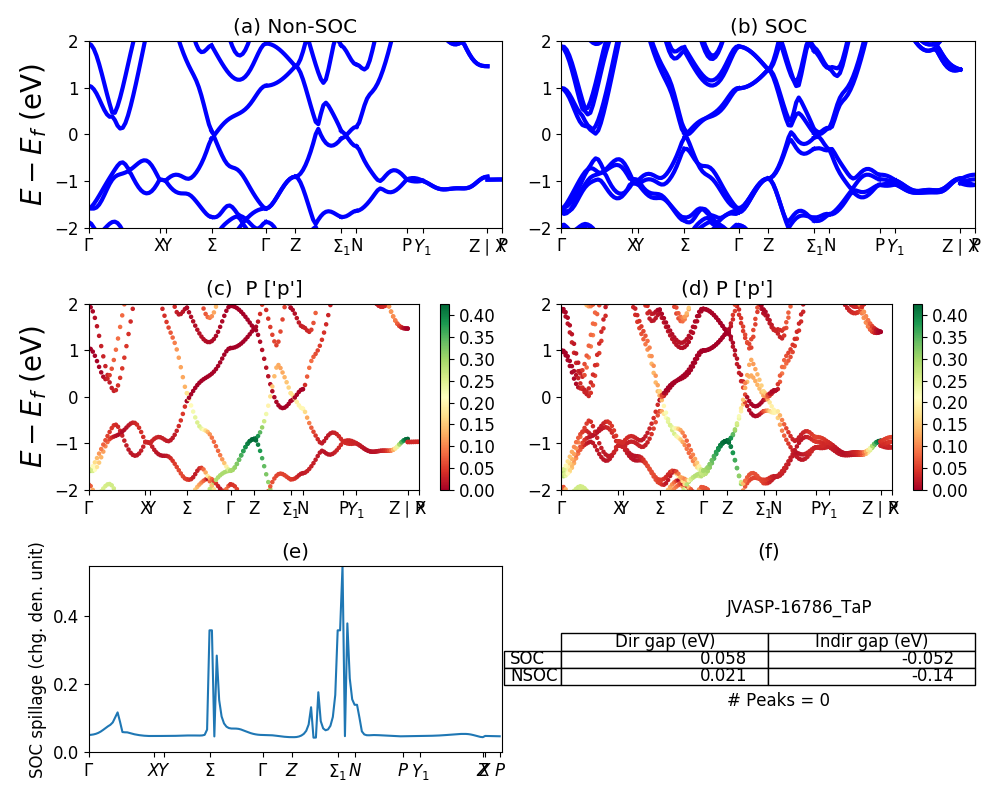


*Fig. S4 a) Non-SOC and b) SOC band structures of TaP (JVASP-16786,* *https://www.ctcms.nist.gov/~knc6/jsmol/JVASP-16786). c) non-SOC and d) SOC projected band structures, projecting onto P-p orbitals. e) Spillage, as a function of k. f) Table of bandgaps and spillage information. TaP is an example Weyl semi-metal.*


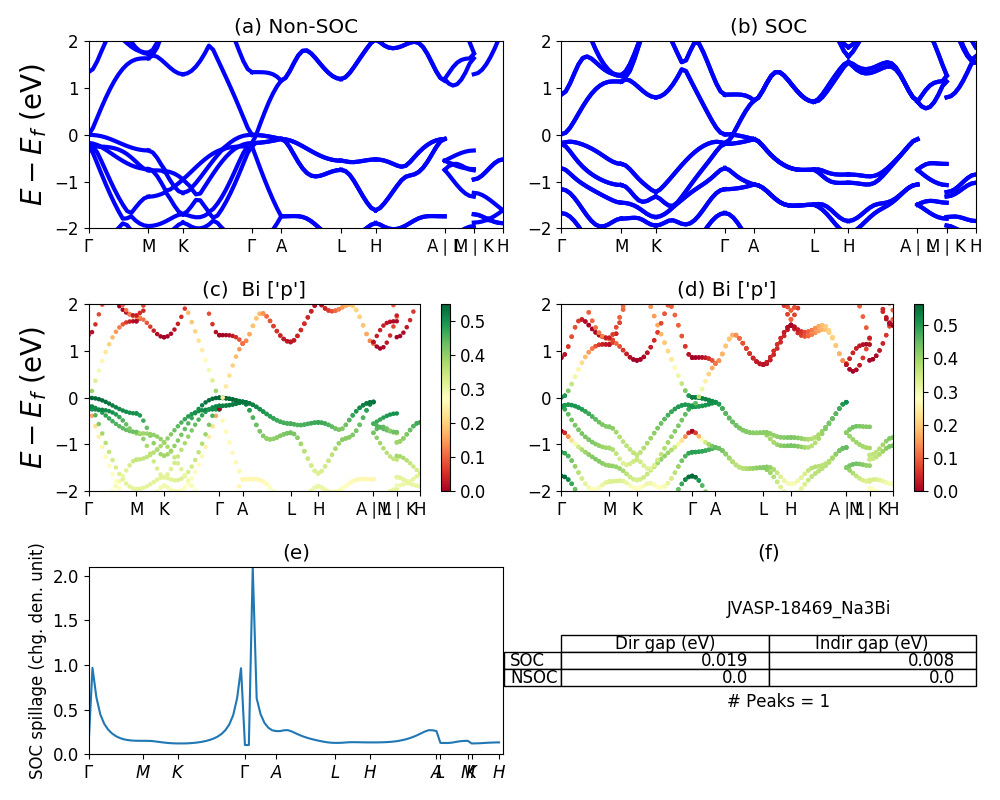


*Fig. S5 a) Non-SOC and b) SOC band structures of Na_3_Bi (JVASP-18469,* *https://www.ctcms.nist.gov/~knc6/jsmol/JVASP-18469). c) non-SOC and d) SOC projected band structures, projecting onto Bi-p orbitals. e) Spillage, as a function of k. f) Table of bandgaps and spillage information. Na_3_Bi is an example Dirac semimetal.*


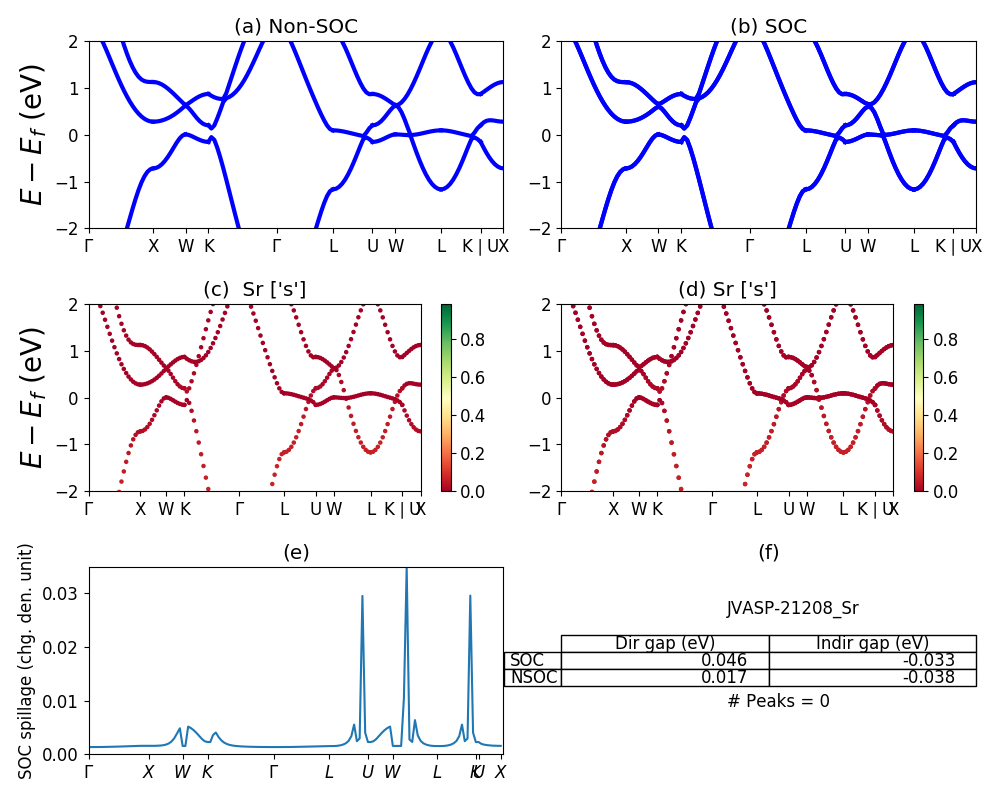


*Fig. S6 a) Non-SOC and b) SOC band structures of Sr (JVASP-21208* *https://www.ctcms.nist.gov/~knc6/jsmol/JVASP-21208). c) non-SOC and d) SOC projected band structures, projecting onto Sr-s orbitals. e) Spillage, as a function of k. f) Table of bandgaps and spillage information. Sr is an example of trivial metal. The spillage value is very low for trivial metals.*


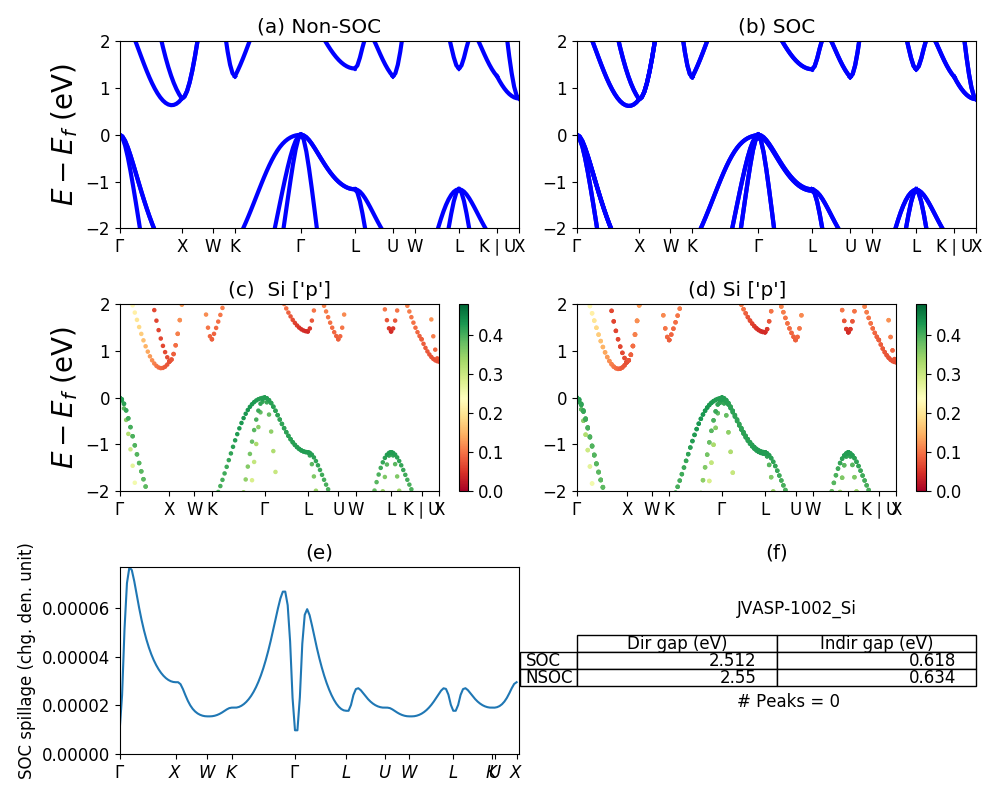


*Fig. S7 a) Non-SOC and b) SOC band structures of Silicon (JVASP-1002, https://www.ctcms.nist.gov/~knc6/jsmol/JVASP-1002). c) non-SOC and d) SOC projected band structures, projecting onto Si-p orbitals. e) Spillage, as a function of k. f) Table of bandgaps and spillage information. The spillage is almost zero for trivial semiconductors.*


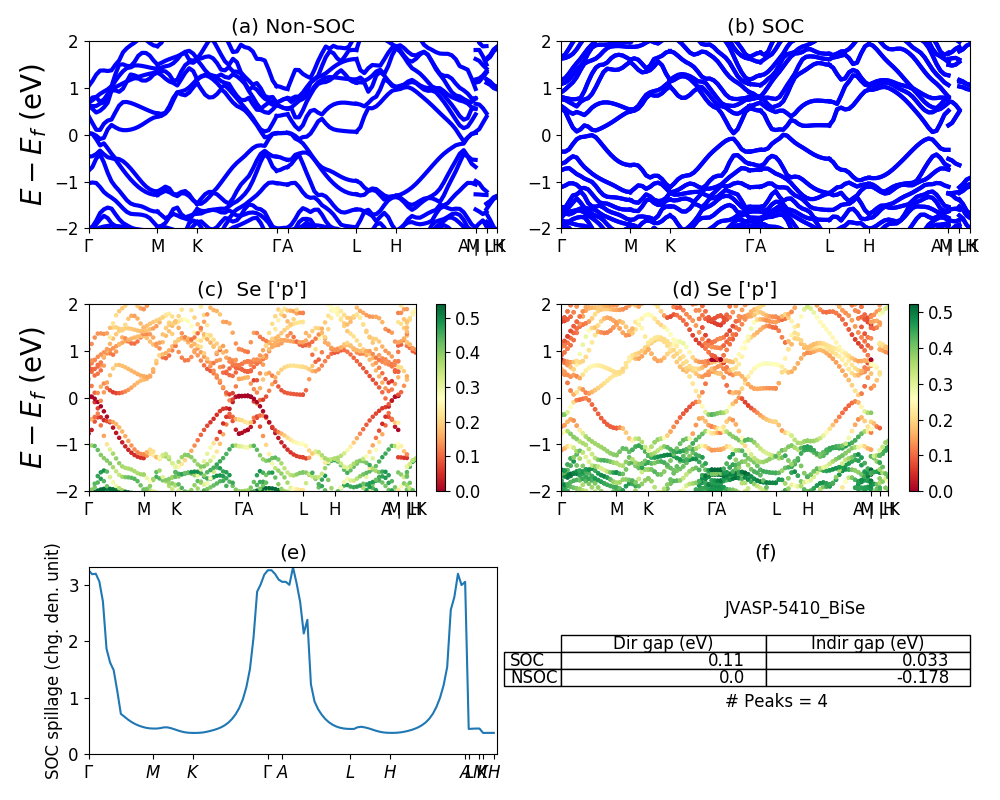


*Fig. S8 a) Non-SOC and b) SOC band structures of Silicon (BiSe, JVASP-5410, https://www.ctcms.nist.gov/~knc6/jsmol/JVASP-5410). c) non-SOC and d) SOC projected band structures, projecting onto Se-p orbitals. e) Spillage, as a function of k. f) Table of bandgaps and spillage information. The bands are inverted along the entire Г-A line, which is also reflected in the spillage.*


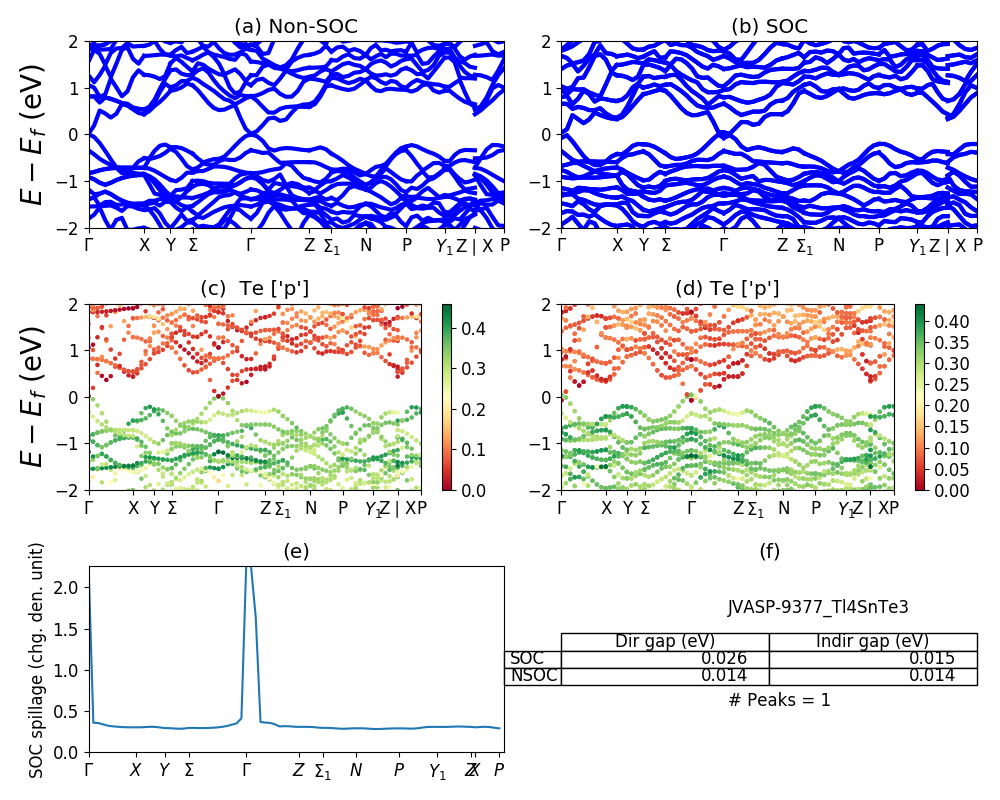


*Fig. S9 a) Non-SOC and b) SOC band structures of Silicon (Tl_4_SnTe_3_, JVASP-9377, https://www.ctcms.nist.gov/~knc6/jsmol/JVASP-9377). c) non-SOC and d) SOC projected band structures, projecting onto Te-p orbitals. e) Spillage, as a function of k. f) Table of bandgaps and spillage information.* *Tl8Sn2Te6 (JVASP-9377) does have a SOC-induced band inversion at Г, however, more Wannier-analysis reveals that the resulting band structure is topologically trivial. False positive examples like this one are very rare.*

Table S1 K-point dependence of spillage value for Bi_2_Se_3_ ($R\bar{3}m$). There is a negligible change in spillage value with the increase in k-point density.

| k-points (per reciprocal atom) | Spillage |
| --- | --- |
| 1 | 2.19 |
| 50 | 2.19 |
| 100 | 2.10 |
| 500 | 2.098 |
| 1000 | 2.098 |


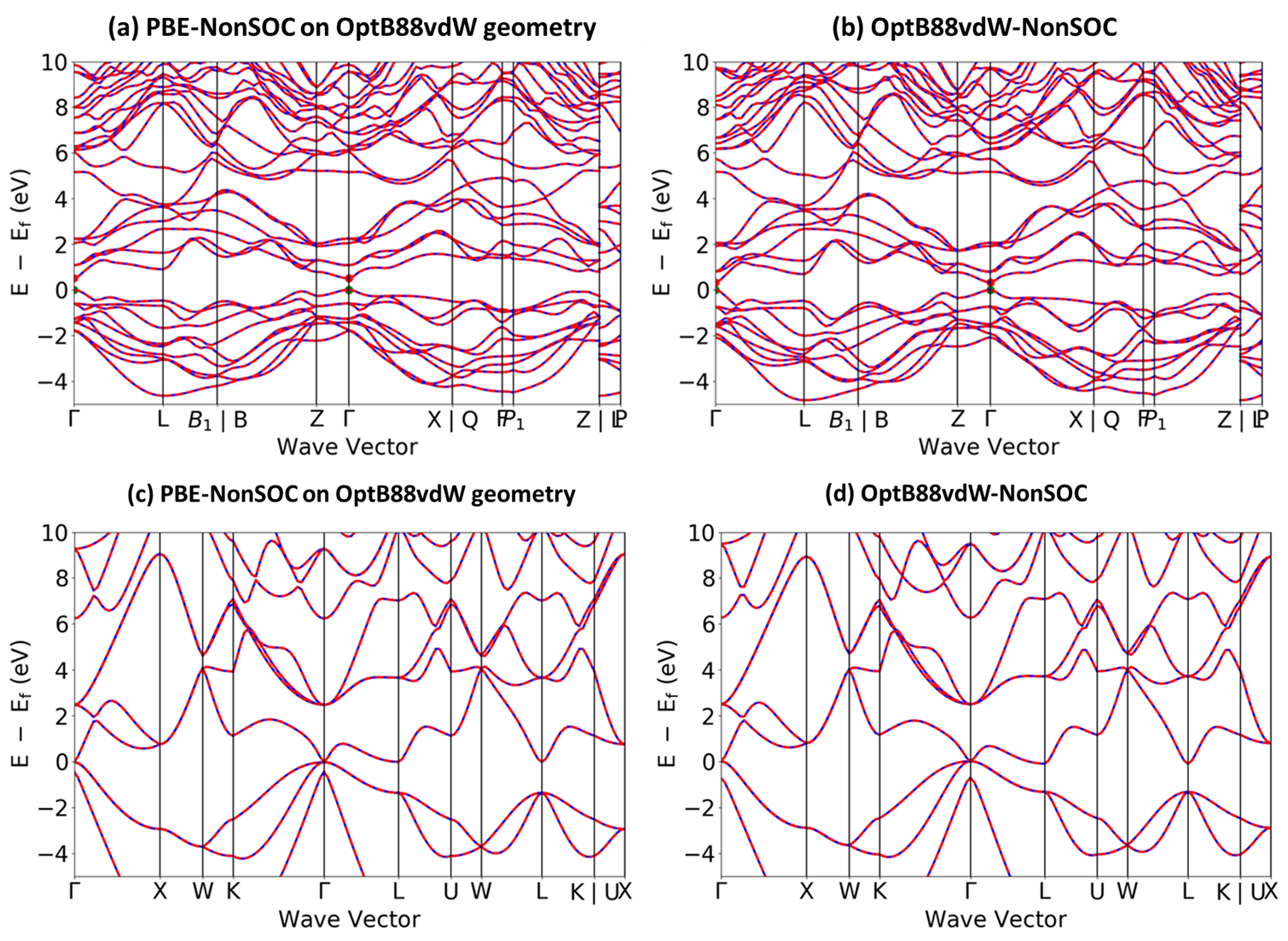


*Fig. S10 Comparison of non-spin-orbit coupling bandstructures on same OptB88vdW relaxed geometry using PBE and OptB8vdW functionals for Bi2Te3 (a and b; topologically non-trivial) and Ge (c and d topologically trivial).*
